# Adaptive neuro fuzzy inference system modeling of *Synsepalum dulcificum* L. drying characteristics and sensitivity analysis of the drying factors

**DOI:** 10.1038/s41598-022-17705-y

**Published:** 2022-08-02

**Authors:** Oladayo Adeyi, Abiola John Adeyi, Emmanuel Olusola Oke, Oluwaseun Kayode Ajayi, Seun Oyelami, John Adebayo Otolorin, Sylvester E. Areghan, Bose Folashade Isola

**Affiliations:** 1 Department of Chemical Engineering, Michael Okpara University of Agriculture, P.M.B 7267, Umudike, Abia State Nigeria; 2 Department of Mechanical Engineering, Ladoke Akintola University of Technology, P.M.B 4000, Ogbomoso, Oyo State Nigeria; 3 Department of Mechanical Engineering, Obafemi Awolowo University Ile Ife, Ife, Nigeria; 4 Department of Mechanical Engineering, Osun State University, Osogbo, Nigeria; 5 Forestry Research Institute of Nigeria, Ibadan, Nigeria

**Keywords:** Chemical engineering, Mechanical engineering

## Abstract

The requirement for easily adoptable technology for fruit preservation in developing countries is paramount. This study investigated the effect of pre-treatment (warm water blanching time—3, 5 and 10 min at 60 °C) and drying temperature (50, 60 and 70 °C) on drying mechanisms of convectively dried *Synsepalum dulcificum* (miracle berry fruit—MBF) fruit. Refined Adaptive Neuro Fuzzy Inference System (ANFIS) was utilized to model the effect and establish the sensitivity of drying factors on the moisture ratio variability of MBF. Unblanched MBF had the longest drying time, lowest effective moisture diffusivity (EMD), highest total and specific energy consumption of 530 min, 5.1052 E−09 m^2^/s, 22.73 kWh and 113.64 kWh/kg, respectively at 50 °C drying time, with lowest activation energy of 28.8589 kJ/mol. The 3 min blanched MBF had the lowest drying time, highest EMD, lowest total and specific energy consumption of 130 min, 2.5607 E−08 m^2^/s, 7.47 kWh and 37 kWh/kg, respectively at 70 °C drying temperature. The 5 min blanched MBF had the highest activation energy of 37.4808 kJ/mol. Amongst others, 3—gbellmf—38 epoch ANFIS structure had the highest modeling and prediction efficiency (R^2^ = 0.9931). The moisture ratio variability was most sensitive to drying time at individual factor level, and drying time cum pretreatment at interactive factors level. In conclusion, pretreatment significantly reduced the drying time and energy consumption of MBF. Refined ANFIS structure modeled and predicted the drying process efficiently, and drying time contributed most significantly to the moisture ratio variability of MBF.

## Introduction

*Synsepalum dulcificum* (Sapotaceae) fruit known as miracle berry fruit (MBF) is an evergreen shrub that is native to West African tropical region^1^. The plant is known as Agbayun amongst the Yorubas of southwestern Nigeria^2^. It is of Sapotaceae family and grows to about 2–5 m high. The plant is hairless with smooth leaves that measured up to 0.05–0.1 m in length and 0.02–0.031 m in width; it has thin branches that clustered at the apex of the branch-lets^3^. *S. dulcificum* is of white flowers and its fruits measured around 0.0200–0.0205 m long^4^. The fruit is of high value to Yorubas due to its usefulness as a sweetener and because it is an ingredient in phyto-medicine concoction making^5^. When the fruit is licked, it deposits an active glycoprotein component called miraculin on the tongue. The action of miraculin is such that it binds to the tongue’s sweet receptor cells and suppresses the brain’s feedback of a sour taste by activating the sweet receptors hence resulting in a perception of sweet taste^4^.

Despite the high value placed on MBF in Nigeria, the poor post harvest plans for the highly perishable fruit is enabling the usually experienced shortages in the off-season production periods. Consequently, enormous quantity of MBF are wasted during the peak production season because of bountiful yield that meets with poor preservation plans; thereby forcing farmers to sell the harvests locally and at any cost possible. Therefore, studies concerning preservation, storage, and reuse of MBF, which are presently unfounded in the literature are crucial to enhance its utilization. To lessen these study gaps, this study proposed the application of practical, effective, and easy to implement drying technology for the preservation of MBF in a developing country like Nigeria where MBF grows, thereby creating a partway for the commercialization of dried MBF products.

Drying is a widely acceptable preservation technique for fruits and vegetables. It aids physical and chemical changes in food products and therefore reduces water activities, prevents microbial and enzymatic activities that otherwise encourage spoilage^6^. In a typical drying process, the dryer supplies the product with higher heat energy than ambient, and as the heat energy increased, the relative humidity of the drying air decreases. The reduced relative humidity decreases the air density and increases the moisture removal capability of the drying air as it moves over the product undergoing drying. At high temperature and low relative humidity, the vapour pressure of the product increases, thereby enabling the moisture migration from the internal part or core to its surface^7^ and to the environment.

Generally, the efficiency of the drying process and quality of the dried product are dependent on the chosen drying factors^8^. These requirements had led researchers to investigate several pre-treatment methods and dryer type for different fruits and vegetables. Considering the effect of pre-treatment of fruit and vegetables prior to drying for instance, Kaveh et al.^9^ established that pre-treating blackberry fruits with solution of ascorbic acid, blanching in hot water, blanching in microwave, and sonication prior to convective drying reduced specific energy consumption, drying time, colour change and shrinkage. Rojas et al.^10^ also concluded that pre-treatment with ethanol prior to apple fruit ultrasound-assisted convective and convective drying improved the moisture mass transfer rate, with reduction in shrinkage and rehydration capacity. Brar et al.^11^ as well showed that ascorbic acid chemical solution, potassium metabisulfite for 1 min at 40 °C and citric acid had significant decreased effect on the time of apple fruit drying. Tunde-Akintunde et al.^12^ concluded that hot water, steam, palm oil/water and groundnut/water blanching significantly reduces the drying rate of bell pepper. The literature review showed that pre-treatment prior to drying have profound positive effect on the efficiency of the drying process which is a strong considerable point for industrial commercialization.

Pre-treatment can be achieved through chemical (liquid and gas phase) or physical (thermal and non-thermal) method^13^; however, the clamour and government legislation favouring green processing and green products, justifies the utilization of physical pre-treatment method on fruit and vegetables. A simply achieved but effective physical pre-treatment method is thermal blanching. It is the process of heating the agro-produce rapidly to a predetermined temperature, holding it at that temperature over a choice resident time, followed by cooling or immediate subsequent processing^14^. Thermal blanching inactivates enzymes, significantly reduces the drying rate, improves the product quality, eliminates pesticide residues and toxic constituents, eliminates the air in products tissues, lessens microbial load amongst others^15^. Thermal blanching is techno-economically suitable to industrial or commercial applications. It is usually done at high-temperature-low resident time or low-temperature-high resident time to prevent tissue disruption or breakdown^14^. However, it is believed that low temperature blanching can minimize process resources amongst other benefits. Jeet et al.^16^ employed warm water (60 °C for 10 min) pre-treatment prior to drying of unripe banana slices. The choice of warm water pre-treatment is usually aimed at mitigating the usually experienced extreme loss in firmness of tender textured agro-produce after hot water blanching (70–100 °C)^13^. Presently, reports on warm water thermal blanching are still nascent and therefore forms a focus for this study.

Also considering the effect of dryer type for fruit and vegetable drying purposes, microwave-drying technique involving volumetric heating and internal vapor generation adopted by Demiray et al.^17^ shortens the drying time of onion when compared to pure convective drying method. Furthermore, the convective dryer has been reported to be less effective with drying time (leading to inefficient use of energy) and slightly decrease product quality (color and vitamin C amongst others) when compared to modern drying technologies like microwave, vacuum, infrared, hybrid ovens and others^18^. However, and despite the notable deficiencies of convective drying, the technique remains the most practicable method of drying especially in developing countries where cost of drying facility installation, operation, and maintenance requirements translate to a big challenge. The convective dryer is cheap to install, easily scaled for varied throughput, easily operated, and maintained. Therefore, utilization of convective drying method is still relevant despite the availability of competitive alternatives.

Modelling is an important endeavour for in-depth understanding, prediction, monitoring, rectification, controlling, alteration and redesigning of the process and equipments^8^. The thin layer models including empirical, theoretical, and semi-theoretical equations have been mostly utilized to analyze and explain drying processes in the last decades^9^. However, recent reports are suggesting the adoption of machine learning and artificial intelligent methods because of their perceived improved accuracies when compared to thin layer models. For instance, Yazdani^19^, Kaveh et al.^9^ and Sakar et al.^20^ applied artificial neural networks (ANN), Adaptive Neuro Fuzzy Inference System (ANFIS) and Gauss Process Regression Based Model (GPR), respectively, to drying of pineapple, orange and sweet potatoes for process modelling, prediction and optimization. Intelligent methods perform best when their structure is optimized before utilized for dataset approximation; however, most literature relied on subjective trial-and-error approach to specify the intelligent method’s structure and this can limit the effectiveness of the tool. More also, the investigation of optimum ANFIS structure (within given structure parameters) to drying data modelling is few in the literature. In addition to predictive modelling, the establishment of sensitivity analysis for a process is necessary to provide detailed information for decision-making^21^. Sensitivity analysis is a conscious platform for process determination and operation and in today’s knowledge advancement; the theoretical methods have been well developed such that it becomes intellectually short-changed to perform modelling without sensitivity analysis^22^. Literature review showed that sensitivity analysis of drying process with ANFIS is currently a gap in study.

This work contributes to knowledge by investigating the material-processing-product relationship that is specific to convectively dried warm water blanched MBF, represents the drying characteristics with an optimized ANFIS structure and investigate the sensitivity of the drying kinetics to drying factors using the optimized ANFIS structure. To the best of our knowledge, these are present gaps in the literature and their elucidation will form a partway to specific equipment, process and controller design and development for a potential MBF’s drying process industrial commercialization and also forms a precursor to MBF preservative plan that will contribute to food security especially in developing countries. The specific objectives were to (a) study the effect of physical pre-treatment (warm water blanching time—3, 5 and 10 min at 60 °C) and drying temperatures (50, 60 and 70 °C) at constant air velocity (1.2 m/s) on the drying characteristics [drying time (min), effective moisture diffusivity (m^2^/s), activation energy (kJ/mol), total energy consumption (kWh) and specific energy consumption (kWh/kg)] of convectively dried MBF (b) optimize the ANFIS network structure to model and predict the drying kinetics of MBF and (c) investigate the sensitivity of moisture ratio to the drying factors (pre-treatment type, drying temperature and drying time) using the optimized ANFIS structure.

## Materials and methods

### Raw material

In this study, the experimental research and field studies on plants, including the collection of MBF, complies with relevant institutional, national, and international guidelines and legislation. Freshly harvested MBF were sourced from a farmland in Ogbomoso Oyo State Nigeria after securing consent from the farmer who owns the farmland. The fruits were harvested, rinsed with distilled water, transported to the laboratory in a closed glass bottle and stored in a refrigerator (Heier thermocool, model 4523, UK) at a temperature of less than 4 °C. Prior to the drying experiment, MBF samples were allowed to thaw at room temperature (26–29 °C) for 1 h. The measured (using vernier calliper) approximate dimension of the MBF used in this study (considering 10 randomly selected samples) were; mass of 3.10 g, diameter of 0.013 m and length of 0.018 m.

### Pre-treatments

Warm water blanching pre-treatment of MBF samples were achieved by dipping samples in warm water (60 °C) for 3, 5 and 10 min, respectively in accordance with the method of Taiwo and Adeyemi^23^. At the completion of the specified resident time in the warm water medium, the samples were removed and wiped dry with tissue paper. The samples were then allowed to attain room temperature by placement on aluminium foil and exposure to air. Thereafter, the samples were subjected to specific drying procedure as described in the next sub-section.

### Drying procedure

The contribution of warm water blanching pre-treatment at different dipping times and different drying temperature on the drying characteristics of MBF were determined. The differently pre-treated MBF were dried in a convective hot air oven (SG-90526—model, Stangas—company, Italy—country) as previously used by the same authors at nutritive quality preserving temperatures of 50, 60, and 70 °C, and at a constant dryer air velocity of 1.5 m/s. The ambient air temperature varied between 28 and 33 °C. At each selected drying temperature, the dryer was allowed to run for 30 min so that a uniform temperature state throughout the drying chamber was achieved before the introduction of selected experimental MBF samples. In each drying run, an approximately 200 g of pre-treated MBF were spread evenly on a tray and in a single layer within the steady state dryer. The instantaneous weights of the sample undergoing drying were determined firstly at 10 min interval during the early stages of the drying process and later at 30 min interval at the later stages of the drying process. This was achieved by discontinuously weighing the sample on digital weighing balance (± 0.01 g precision) and repeated severally until a constant MBF sample weight was observed. Experiments were done in three replicates for the purpose of data integrity and the mean of the replicates were used to describe the drying characteristics of the MBF samples. The initial moisture content of the un-blanched, 3 min, 5 min and 10 min warm water blanched samples were determined to be 10.91%, 11.03%, 11.14% and 11.43%, respectively by using the oven dried method at 105 °C for 24 h^24,25^.

### Drying indicators

The drying characteristics of MBF were evaluated by determining the moisture ratio, effective moisture diffusivity (EMD), activation energy, and energy consumption. In addition, ANFIS network structure was refined for the multidimensional predictive modelling of the drying process; and applied for the sensitivity analysis of moisture ratio to the individual and interaction of drying process factors used in this study.

#### Moisture ratio

The moisture content is a subset of moisture ratio. It was therefore calculated first using Eq. (1) as employed by Bousselma et al.^26^.

1 $${\text{M}}_{{\text{c}}} = \frac{{{\text{W}}_{{\text{w}}} - {\text{ D}}_{{\text{w}}} }}{{{\text{D}}_{{\text{w}}} }} \times 100$$

where M_c_ is the moisture content (d.b), W_w_ is the sample wet weight (g), and D_w_ is the samples dry weight (g).

Thereafter, the moisture ratio throughout drying was calculated with Eq. (2)

2 $${\text{MR}} = \frac{{{\text{M}}_{{\text{t}}} - {\text{ M}}_{{\text{e}}} }}{{{\text{M}}_{{\text{o}}} - {\text{M}}_{{\text{e}}} }}$$

where MR represents the moisture ratio, M_t_ represents the moisture content (g water/g dry matter) throughout the drying time, M_o_ represents the initial moisture content (g water/g dry matter), and M_e_ represents the equilibrium moisture content (g water/g dry matter) of the MBF.

#### Effective moisture diffusivity

EMD is an important drying characteristic that describes the moisture transportation rate from the sample’s internal component to the sample’s surface^27^. Previous studies by Motevali et al.^28^ and Sarvestani et al.^29^ showed that moisture transport could be investigated by the use of Fick’s second law of diffusion. Sarvestani et al.^29^ used Eq. (3) as the Fick’s second law of diffusion for sphere shaped sample like MBF.

3 $$\frac{1}{r} \frac{\partial }{\partial r} \left( {r^{2} \frac{\partial M}{{\partial r}}} \right) = \frac{1}{{D_{eff} }} \frac{\partial M}{{\partial t}}$$

The solution to Eq. (3) is represented by Eq. (4) in one-dimensional spherical system.

4 $${\text{MR }} = \frac{{M_{t} {-} M_{e} }}{{M_{o} - M_{e} }} = \frac{6}{{\pi^{2} }}\mathop \sum \limits_{n = 0}^{\infty } \frac{1}{{n^{2} }}\exp \left( { - \frac{{n^{2} \pi^{2} D_{eff} t}}{{r^{2} }}} \right)$$

At long drying time (n = 1), Eq. (4) reduces to Eq. (5)

5 $${\text{In MR }} = {\text{ In}}\left( {\frac{6}{{{\uppi }^{2} }}} \right) - {\text{In}}\left( {\frac{{{\uppi }^{2} {\text{ D}}_{{{\text{eff}}}} {\text{t}}}}{{{\text{r}}^{2} }}} \right)$$

where D_eff_ is the representation of effective moisture diffusivity (m^2^/s), r is the representation of radius of the fresh fruit and t is the representation of time (min). The EMD was then determined by applying the slope method where the plot of Ln MR versus t gives a straight-line curve with slope K_L_ as represented in Eq. (6).

6 $${\text{K}}_{{\text{L}}} = \frac{{{\text{D}}_{{{\text{eff}}}} {\uppi }^{2} }}{{{\text{r}}^{2} }}$$

#### Activation energy

This refers to the energy required for the initiation of moisture migration from the sample’s core to its surface before being evaporated to the environment. In accordance with the method employed by Motevali et al.^28^, activation energy was established with Arrhenius equation given in Eq. (7).

7 $${\text{D}}_{{{\text{eff}}}} = D_{o } exp\left( {{-}{ }\frac{{{\text{E}}_{{\text{a}}} }}{{{\text{RT}}}}} \right)$$

where E_a_ represents the activation energy (kJ/mol), T represents the absolute temperature (K), R represents the universal gas constant (8.3 kJ/mol), and *D*_*o*_ represents the Arrhenius equation’s pre-exponential factor (m^2^/s). Simply, Ln ${\text{D}}_{{{\text{eff}}}}$ was plotted against the T^−1^ to derive the activation energy.

#### Total and specific energy consumption

Adopting the method of Tunde-Akintunde^30^ and İsmail et al.^31^, the energy consumption was determined. The total and specific energy consumption were determined using Eqs. (8) and (9), respectively.

8 $${\text{E}}_{{\text{t}}} = {\text{Av}}{{\uprho }}_{{\text{a}}} {\text{c}}_{{\text{a}}} \Delta{\text{TD}}_{{\text{t}}}$$

9 $${\text{E}}_{{\text{s}}} = \frac{{{\text{E}}_{{\text{t}}} }}{{{\text{W}}_{0} }}$$

where ${\text{E}}_{{\text{t}}}$, ${\text{E}}_{{\text{s}}}$, ${\text{A}},\;{\text{v}},\;{\uprho }_{{\text{a}}} ,\;{\text{c}}_{{\text{a}}} ,\;{\Delta T},\;{\text{D}}_{{\text{t}}}$ and W_0_ are the total energy required for drying (KWh), specific energy consumption (KWh/kg), the tray area (m), air velocity (m/s), the air density (kg/m^2^), specific heat capacity of the drying air (kJ/kg °C), temperature difference (°C), time of drying (h) and initial weight (kg), respectively.

#### ANFIS modeling

**Figure 1:**
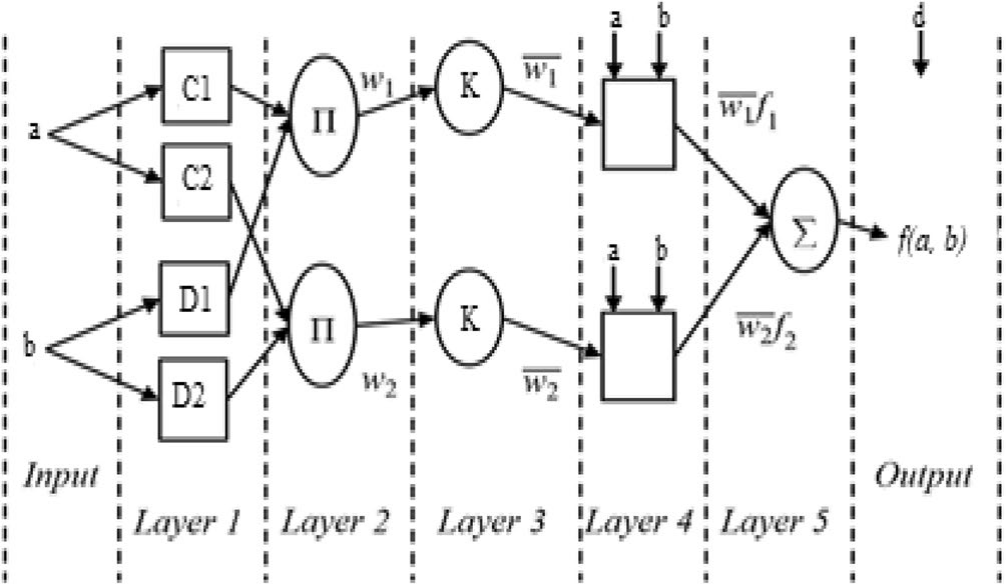
The ANFIS network structure.

ANFIS is a representation of Sugeno fuzzy inference system. It is a hybridization of ANN and Fuzzy logic (FL) and models multiple input data relating to a single output data^27^. The FL part of ANFIS matches or maps the input data characteristics to input membership functions, input membership function to rules, rules to a set of output data characteristics, output characteristics to output membership function and the output membership function to the output. The outline of the membership function is proportional to values that are adjustable in other to change the outline of the membership function^32^. In furtherance, the ANN part of ANFIS plays the role of automatically adjusting the parameters of the FL membership function and thereby optimizing its structure in the process. ANN achieves the membership function structural adjustment using a back propagation algorithm or with a least square approximation type of method. The parameters associated with membership functions change through the learning process. FIS learn and develops rules from data during a training process for approximation. A generalized model of ANFIS structure consisting of two factors contribution or inputs—a and b is represented in Fig. 1.

The rules of a typical ANFIS structure is represented in Eqs. (10–13) as follows;

10 $${\text{Rule 1:}}{\text{ If a is C}}_{{1}} {\text{and b is D}}_{{1}} ,{\text{ then f}}_{{1}} = {\text{ p}}_{{1}} {\text{a }} + {\text{ q}}_{{1}} {\text{b }} + {\text{ r}}_{{1}}$$

11 $${\text{Rule 2:}}{\text{ If a is C}}_{{2}} {\text{and b is D}}_{{2}} ,{\text{ then f}}_{{2}} = {\text{ p}}_{{2}} {\text{a }} + {\text{ q}}_{{2}} {\text{b }} + {\text{ r}}_{{2}}$$

12 $${\text{Rule 3:}}{\text{ If a is C}}_{{3}} {\text{and b is D}}_{{3}} ,{\text{ then f}}_{{3}} = {\text{ p}}_{{3}} {\text{a }} + {\text{ q}}_{{3}} {\text{b }} + {\text{ r}}_{{3}}$$

13 $${\text{Rule 4:}}{\text{ If a is C}}_{{4}} {\text{and b is D}}_{{4}} ,{\text{ then f}}_{{4}} = {\text{ p}}_{{4}} {\text{a }} + {\text{ q}}_{{4}} {\text{b }} + {\text{ r}}_{{4}}$$

In Fig. 1, the input layer consists of two inputs ‘a’ and ‘b’. The input Layer 1 as represented by Eq. (14) is the crisp inputs for the system under consideration, ${\text{O}}^{1}$ is a representation of the layer, and *i* is the representation of ANFIS node.

14 $${\text{O}}_{i}^{1} = \, \left( {\text{input factors}} \right)$$

In layer 2, the given crisp input data are conformed into a fuzzy space using a membership function of choice. This process is referred to as fuzzification, hence the layer is known as fuzzification layer. This layer has adaptive nodes as represented in Eq. (15) and (16); μA_i_ and μB_i_ are the representation of the input membership functions.

15 $${\text{O}}_{i}^{2} = \mu {\text{A}}_{{\text{i}}} \;({\text{input}}\;{\text{factor}}\;{1})\quad {\text{for}} = { 1},\;{2}$$

16 $${\text{O}}_{i}^{2} = \mu {\text{B}}_{{\text{i}}} \;({\text{input}}\;{\text{factors}}\;{2})\quad {\text{for}} = { 3},\;{4}$$

Layer 3 is referred to as the firing strength layer of the structure. This layer determines the product of the degrees to which the input factors fits into the membership function of choice and it is depicted in Eq. (17).

17 $${\text{O}}_{i}^{3} = wi = \mu {\text{A}}_{{\text{i}}} \;({\text{input}}\;{\text{factor}}\;{1}) \, \times \mu {\text{B}}_{{\text{i}}} \;({\text{input}}\;{\text{factor}}\;{2}), \ldots i = {1},\;{2},\;{3},\;{4}$$

Layer 4 is termed as the normalization layer. The ratio of the firing strength of each rule is calculated with respect to the sum of the firing strengths of all the rules. The layer also has fixed nodes as depicted in Eq. (18).

18 $${\text{O}}_{i}^{4} = w^{\prime} = wi/w{1} + w{2}, \ldots = { 1},\;{2}$$

Layer 5 is termed as defuzzification layer, it is represented in Eq. (19). The layer consists of the consequent parameters of the fuzzy rules. The neurons in this layer are very connected to the normalization neuron.

19 $${\text{O}}_{i}^{5} = w^{\prime}_{i} f_{i} = w^{\prime}(p_{i} x + q_{i} y{ + }r_{i} ),\quad i = {1},\;{2}$$

Layer 6 produces the total output for each input in the fuzzy space, which equals the sum of inputs in layer 5. It is represented in Eq. (20).

20 $${\text{O}}_{i}^{6} = {\text{overall}}\;{\text{output }} = \sum_{i} w^{\prime}_{i} f = \, \sum_{i} w_{i} f_{i} / \sum \, = { 1},\;{2}$$

The type and number of membership function are the two most important parameters that affect the performance of ANFIS network structure for classification, modeling and clustering. In this study, the best-tuned choice of these two parameters was investigated. Effect of four membership functions types (trimf, pimf, gbellmf and gaussmf) and three numbers of each membership function (2, 3 and 5 mf) on the accuracy of the built ANFIS network structure for modeling and prediction of the MBF drying data was investigated. The drying data set were partitioned into training (60%), checking (15%), and testing (25%) for the entire ANFIS model network structure development in conformation with the acceptable standard^33^.

#### Sensitivity analysis

The investigation of the level of contribution or importance of each drying factor (or combination of drying factors) to the performance of the drying process (as measured by a characteristic drying indicator) is crucial for an in-depth understanding of the process and also crucial to making operational decision for the process. Such investigation represents the sensitivity of a process characteristic or performance indicator to the process factors. In this study, the sensitivity of MFB’s moisture ratio during drying to each drying factor (pretreatment type, drying time, and drying temperature) and combination of drying factors were evaluated using ANFIS. The contribution of the individual drying factor (or combination of drying factors) to the prediction accuracy [as measured with root mean square error (RMSE)] of the moisture ratio was utilized as the yardstick to determine the sensitivity. Hence, an individual drying factor (or a specific combination of drying factors) that has lower RMSE contribution to the prediction of moisture ratio is more sensitive than an individual drying factor (or a specific combination of drying factors) that has a higher RMSE contribution to the prediction of moisture ratio.

## Result and discussion

### Effect of warm water blanching pretreatment and drying temperature on the moisture ratio of MBF

**Figure 2:**
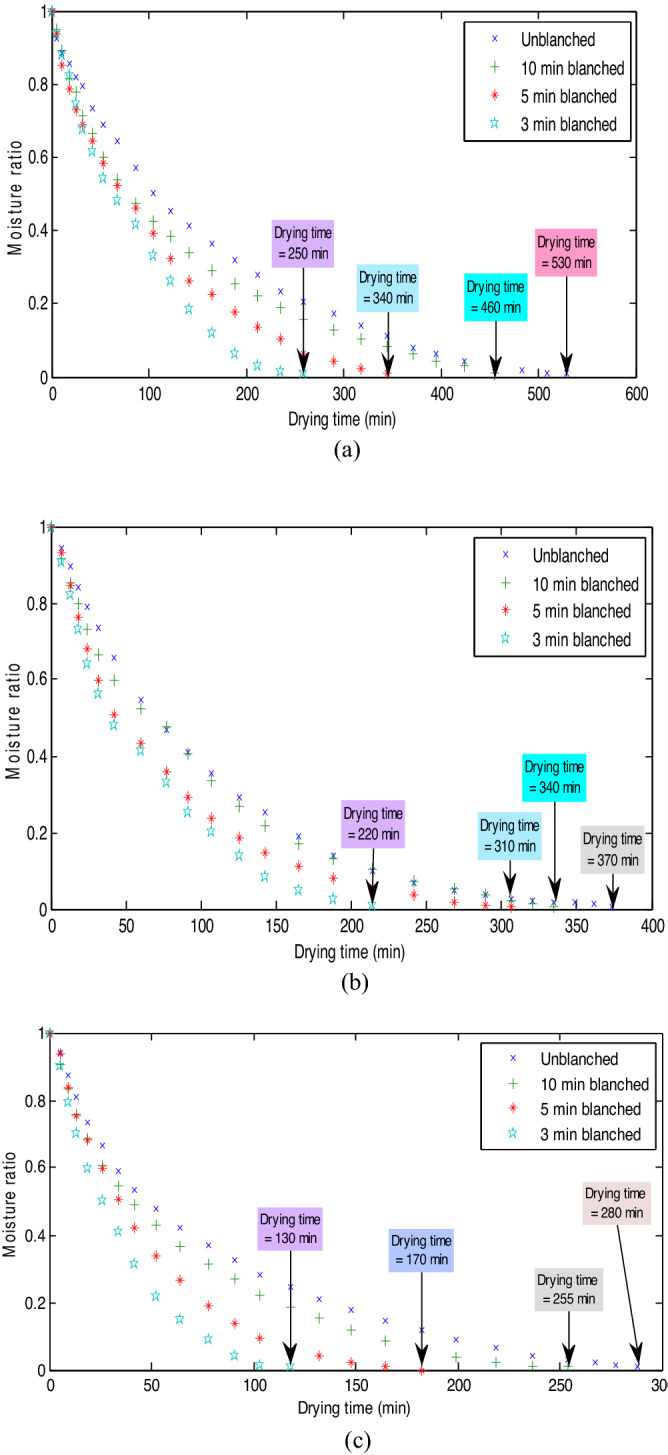
Effect of pretreatment and drying temperature on moisture ratio kinetics at (**a**) 50 °C, (**b**) 60 °C and (**c**) 70 °C.

The effect of pretreatment and drying temperature on the moisture ratio variation of MBF is represented in Fig. 2a–c.

Agricultural products are considered porous in nature^34^, and therefore, when subjected to hot air drying, the heat energy travels through the pores and make the product’s moisture (including surface, hydrate and cell bound waters) content to migrate outwards to the surface from the core and eventually evaporate into the environment. Usually, hot air is dehumidified, energized and is in a state of thirst or disequilibrium. In a bid to revert to its original state, the disequilibrium air strives to absorb moisture while hovering over the agricultural product in the dryer. Hence, the porous characteristics of agricultural product and the action of disequilibrium air on agricultural product ensure the moisture dehydration of the product. The profile in Fig. 2a–c conforms to other reported profiles on the drying of agricultural fruits, vegetables, and crops^35^. The profile showed that MBF drying occurred in three stages at all the tested pretreatment types and drying temperatures. These are the initial fast drying stage (initial linear-like portion of the profile), low drying stage (middle non-linear concave portion of the profile) and lowest drying stage (last linear-like portion of the curve). Generally, these stages are dependent on the moisture content availability and nature of the internal cell structure of the product undergoing drying, however, case hardening also make its contribution especially within the low and lowest drying stages^34^. It is also observed that the drying profile in Fig. 2a–c does not exhibit any constant rate drying period (step profile), instead, the drying took place entirely in the falling drying period (steep profile). This implies that the mechanism of moisture migration in MBF was predominantly diffusion controlled. This observation conforms to the report of Elmas et al.^36^.

Furthermore, Fig. 2a–c showed that untreated MBF sample exhibited the longest drying time followed by 10 min, 5 min and 3 min warm water blanched MBF samples at all selected drying temperatures. The associated long drying time in untreated MBF showed that quick moisture content removal was difficult when compared to the pretreated ones. This may probably be due to its firm natural cell structure (which meant a tight fruit’s moisture to tissue). By implication, the applied pretreatment may have disrupted the firm natural cell structure of MBF and reduced the surface wax too, leading to the crashing down of the drying time significantly. A similar report on the capability of blanching to reduce the drying time was made by Tunde-Akintunde^30^. In addition, Fig. 2a–c also showed that the drying time variation in the pretreated MBF samples followed a definite trend where an increase in pretreatment time led to an increase in drying time in all drying temperatures. This observation may be attributed to the differences in the initial moisture content of the pretreated samples, which increased as the sample’s resident time in the blanching medium increased. The observation can also be explained by a possible increase in the collapse of the cell pore structures while the pretreatment time increased from 3 to 10 min.

In addition, the effect of drying temperature on the drying time is observable in Fig. 2a–c. The increment in drying temperature showed a direct proportionality to reduction in drying time. This can be explained by the fact that increased temperature can quicken the relative humidity reduction of drying air. Therefore, as the drying temperatures increased, the inward heat transfer, outward moisture migration, and surface moisture evaporation of MBF are highly enhanced leading to decreased drying time. Cheng et al.^37^, made the same observation during the drying of cherry tomato; likewise, Abano^38^ made the same observation during the drying of Orange-Fleshed Sweet Potato Slices (*Ipomoea batatas*).

### Effective moisture diffusivity of MBF

**Figure 3:**
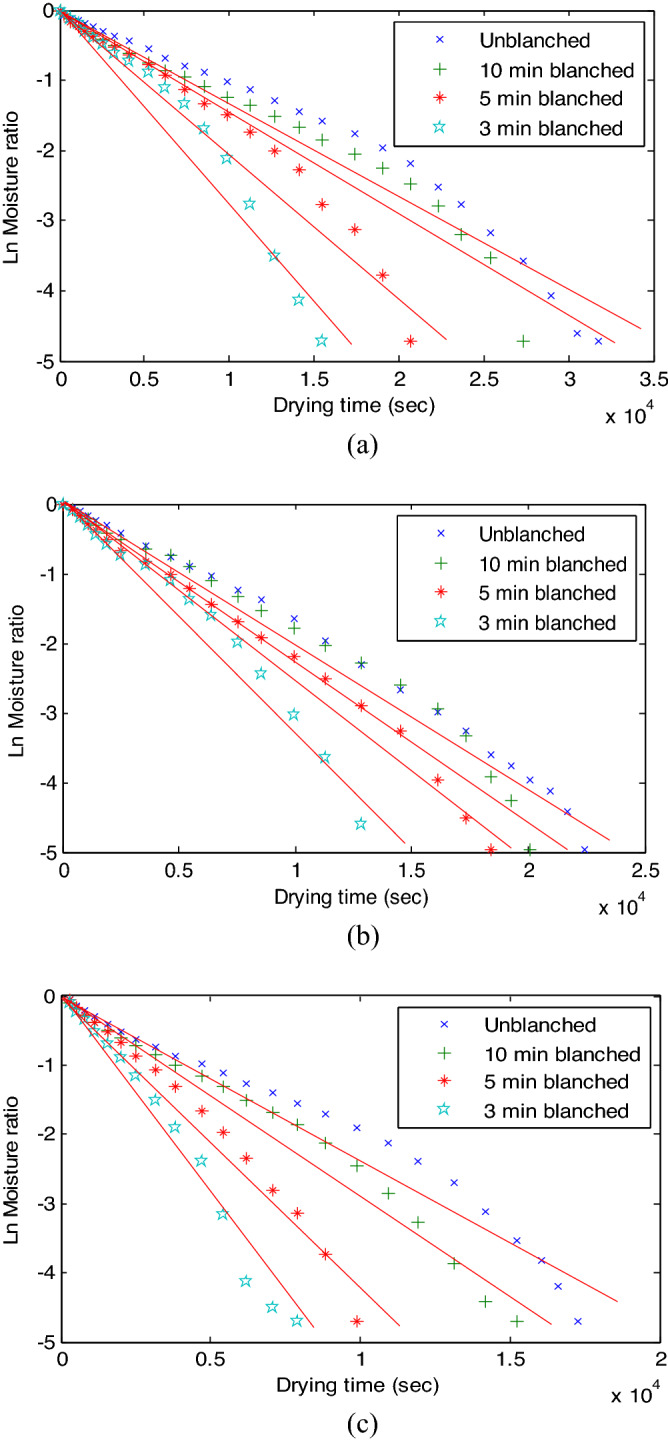
Estimation of the effective moisture diffusivity (**a**) 50 °C, (**b**) 60 °C and (**c**) 70 °C.

The effective moisture diffusivity of the untreated and treated samples of MBF at different drying temperatures were estimated through the graphical method and in connection with Eq. (6) is shown in Fig. 3a–c. Effective moisture diffusivity is an indication of the ease of moisture migration from the sample’s core to its surface.

The calculated effective moisture diffusivity for each MBF samples is shown in Table 1. The table showed that the effective diffusivity ranged from 5.1052 E−09–1.0088 E−08. This conforms to the generally reported range (10^–12^–10^–8^ m^2^/s) of moisture diffusivity in fruit, vegetables, and crops^39^. It can be concluded that the effective moisture diffusivities determined and reported in Table 1 are reliable judging from the high coefficient of determination (R^2^) values of the equation of fit that ranged from 0.9553 to 0.9895.

**Table 1:** Effective moisture diffusivities of MBF.

Sample	Temp. (°C)	Deff (m^2^/s)	Eqn. of fit	R^2^
Unblanched	50	5.1052 E−09	− 0.000134x + 0.170462	0.9600
Unblanched	60	8.3061 E−09	− 0.000205x + 0.162015	0.9895
Unblanched	70	9.5217 E−09	− 0.000235x + 0.086470	0.9685
10 min blanched	50	5.2268 E−09	− 0.000136x + 0.033734	0.9553
10 min blanched	60	8.5898 E−09	− 0.000212x + 0.136115	0.9670
10 min blanched	70	1.1628 E−08	− 0.000287x + 0.086887	0.9766
5 min blanched	50	7.8199 E−09	− 0.000193x + 0.128717	0.9602
5 min blanched	60	1.01 E−08	− 0.000249x + 0.075713	0.9863
5 min blanched	70	1.7706 E−08	− 0.000437x + 0.177591	0.9808
3 min blanched	50	1.1507 E−08	− 0.000284x + 0.275632	0.9540
3 min blanched	60	1.3249 E−08	− 0.000327x + 0.160177	0.9704
3 min blanched	70	2.5607 E−08	− 0.000632x + 0.234027	0.9821

Furthermore, Table 1 showed that unblanched MBF sample dried at 50 °C had the lowest effective moisture diffusivity while the 3 min blanched MBF sample dried at 70 °C had the highest effective moisture diffusivity. The trend of the effective moisture diffusivity showed that it increased with decrease in pretreatment time and increase in drying temperature. These results conform to the observations made in the section concerned with the “Effect of warm water blanching pretreatment and drying temperature on the moisture ratio of MBF”, where increased drying temperature reduced drying time and increased pretreatment time increased the drying time. Therefore, the same mechanism should govern the observations in the two sections.

### Activation energy of MBF

**Figure 4:**
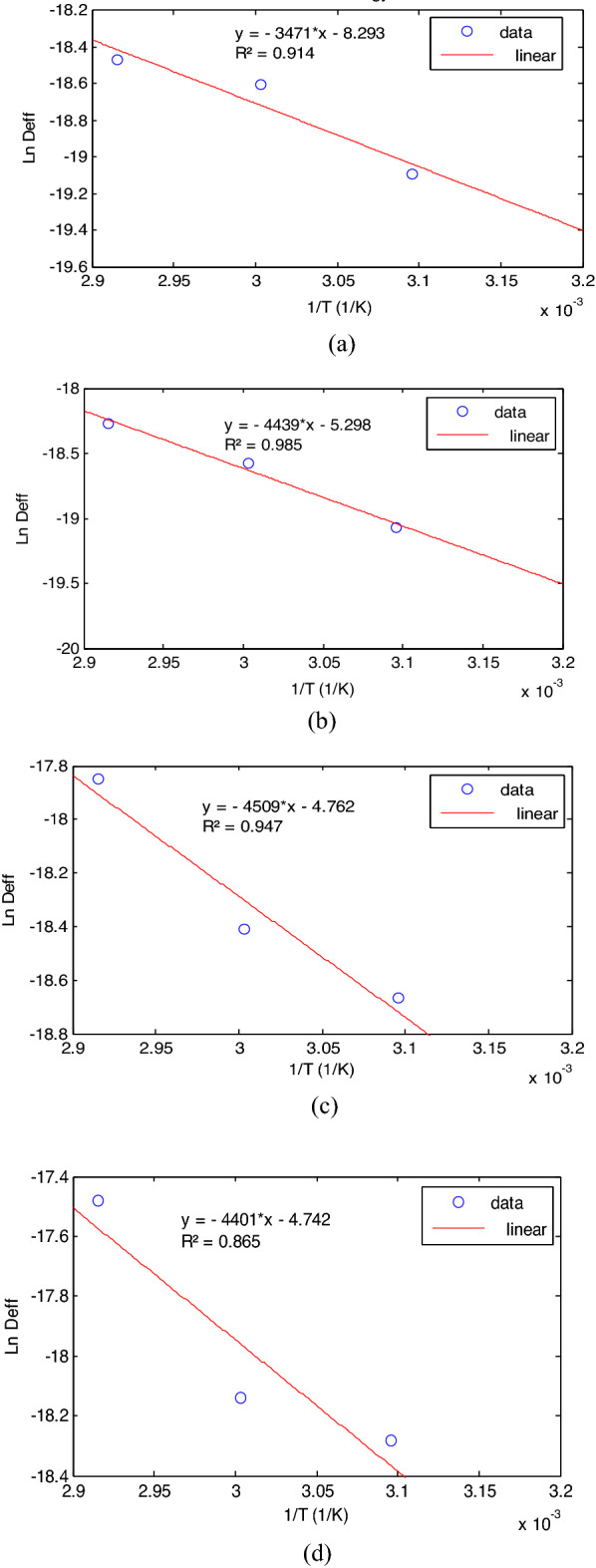
Estimation of the activation energy of (**a**) unblanched, (**b**) 10 min blanched, (**c**) 5 min blanched and (**d**) 3 min blanched.

The activation energy is a representation of the energy utilized to initiate diffusion of moisture in a product and it shows the relationship between effective moisture diffusivity and temperature^27,40^. In this study, the graphical method used for the establishment of activation energies of the samples are depicted in Fig. 4a–d. In line with other reports^41^, the graphical plots were all straight lines and therefore indicates an Arrhenius relationships (effective moisture diffusivity increased with increased temperature). The equation of fit used for the calculation of the activation energies and their respective accuracies (R^2^) were depicted on the Fig. 4a–d. The high R^2^ values showed that the equations are dependable.

Going forward, the calculated activation energy for each sample in this study is represented in Table 2. The obtained activation energy values were within the generally reported range of 12.7–110 kJ/mol for foods, fruits and vegetables^30^. The Table 2 showed that unblanched samples had the least activation energy when compared to the blanched samples despite the unblanched sample’s reported least effective moisture diffusivity in the previous section. This observation may be explained with the fact that, though dissociation of the cell bound moisture within the core of unblanched MBF was easily activated, the naturally strong waxy outer coating of unblanched MBF delayed the crossing of the dissociated moisture from within the cell structure to the surface layer. This also explains the comparably longest drying time exhibited by unblanched MBF sample in the previous section. A close observation where unblanched sample had least activation energy compared to blanched sample was also reported by Cheng et al.^37^ in a study concerning the influence of blanching on the drying characteristics of Cherry Tomato. Table 2 also showed that 5 min blanched sample had the highest activation energy and 3 min blanched sample had the lowest activation energy within the blanched MBF samples. The difference between the activation energies of blanched MBF samples is marginal.

**Table 2:** Activation energy of MBF.

Sample	Activation energy (kJ/mol)
Unblanched	28.8589
10 min blanched	36.9071
5 min blanched	37.4808
3 min blanched	36.5912

### Drying process energy consumption

**Figure 5:**
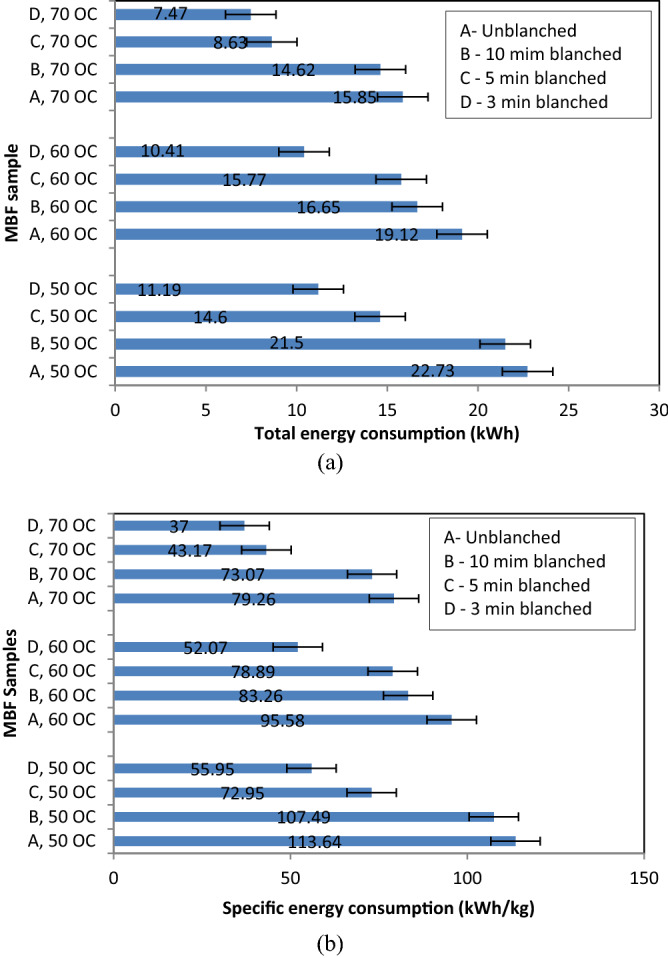
Energy consumption of the drying process.

The energy intensive nature of the convective drying technology necessitates the understanding of its total and specific energy requirement of specific products dried. This understanding enables convective drying process to be conducted economically for optimal gains. The Fig. 5a,b showed the total and specific energy consumed during the drying of unblanched and differently blanched MBF samples in this study.

As represented in Fig. 5a, the highest total energy consumption was observed in unblanched samples dried at 50 °C while the lowest energy consumption was observed in 3 min blanched samples dried at 70 °C. The figure also showed that total energy consumption decreased with decreased temperature and decreased pre-treatment time. Likewise, the specific energy consumption in represented in Fig. 5b takes the same trend as observed in Fig. 5a. The observed total and specific energy consumption seems proportional where samples with higher drying time had higher energy consumption. This observation had been attributed to the cell structure condition and strong waxy surface tissue of MBF that was affected by the applied pretreatment type and the dryer operational temperature that energize the water vapor in MBF during drying. Adeyi et al.^41^ also reported a close result during the drying of Cobra 26 F1 tomato slab in a convective dryer.

### Descriptive statistics of the drying data

The descriptive statistics of the experimental data used for predictive model development in this study is represented in Table 3. The descriptive statistics enables familiarity and understanding of the data of interest.

**Table 3:** Description of the experimental data.

S/N	Description statistics	Drying time	Drying temperature	Pre-treatment	Moisture ratio
1	Mean	123.5249	59.4509	4.4588	0.3931
2	Standard error	7.7200	0.5141	0.2428	0.0204
3	Median	86.9820	60	5	0.3240
4	Mode	0	50	0	1
5	Standard Deviation	123.2785	8.2105	3.8787	0.3259
6	Sample Variance	15,197.6076	67.4139	15.0445	0.1062
7	Kurtosis	0.4528	− 1.5098	− 1.3072	− 1.1870
8	Skewness	1.0760	0.1021	0.3299	0.4425
9	Range	529.4370	20	10	0.9930
10	Minimum	0	50	0	0.0070
11	Maximum	529.4370	70	10	1
12	Sum	31,498.8510	15,160	1137	100.2550
13	Count	255	255	255	255

Amongst other data description given in Table 3, the skewness represents the measure of data inequality around its mean value. The skewness can be positive, negative, or undefined; and in a normal distribution, the tail on either side of the skewness curve is the exact mirror image of one another. Also, the Kurtosis measures the profusion or lack of outliers in relation to a normal data distribution. The kurtosis is regarded as heavily tailed or light tailed depending on the quantity of outliers in a data. It should be noted that the Kurtosis is distinct from standard deviation, which quantifies the amount by which data differs from the arithmetic mean. The types of kurtosis include meso, lepto and platykurtic. The other data descriptors including mean, median, mode, standard error, samples variance, standard deviation and others are literal.

The skewnesses in all the data factors (drying time, drying temperature, pretreatment and moisture ratio) are low and positive meaning that the data are normally distributed and has symmetry about its mean in a frequency distribution. Furthermore, the Kurtosis values in the factors are also low. This shows that a larger portion of the data fits to a specific distribution while very few portion deviates. Therefore, the data is uniformly organized.

### ANFIS predictive modeling of MBF drying process

Having had being familiar with the data, ANFIS was trained to develop a network model structure for the process. Such networked model structure is required for process analysis, understanding, redesigning, decision-making, and control. Compared to empirical models, artificial intelligent models are easily adapted for controller design and can deal with multidimensional factors or systems. To determine the best ANFIS network predictive model structure for the drying data in this study, the effect of membership function type and membership function number on the accuracy of the structure was investigated. The result of the investigation is represented in Table 4.

**Table 4:** ANFIS varied structural performance.

S/N	Membership function type	Membership function number	Epoch	R^2^
1	Trimf	2	250	0.8879
2	Trimf	3	392	0.9123
3	Trimf	5	278	0.9136
4	gbellmf	2	60	0.9823
5	gbellmf	3	38	0.9931
6	gbellmf	5	83	0.9917
7	gaussmf	2	153	0.9201
8	gaussmf	3	128	0.9410
9	gaussmf	5	29	0.8923
10	pimf	2	431	0.9127
11	pimf	3	211	0.9726
12	pimf	5	391	0.9812

The table showed that the ANFIS structure developed with three (3) numbers of trimf membership function type was the least efficient (in terms of R^2^ value) while the ANFIS structure developed with five (5) numbers of gbell membership function was the most efficient amongst the 12 combinations of the membership function type—membership function numbers tried. The most efficient ANFIS structure also had the least epoch number. Few epoch numbers are good for limiting the computer memory exhaustion during ANFIS predictive modeling. It should also be noted that increased membership function number increased the complexity of the ANFIS structure thereby elongating the simulation time. This according to Table 4, does not necessarily translate into high performance. Therefore, a too complex or too simple ANFIS network structure should be avoided.

**Figure 6:**
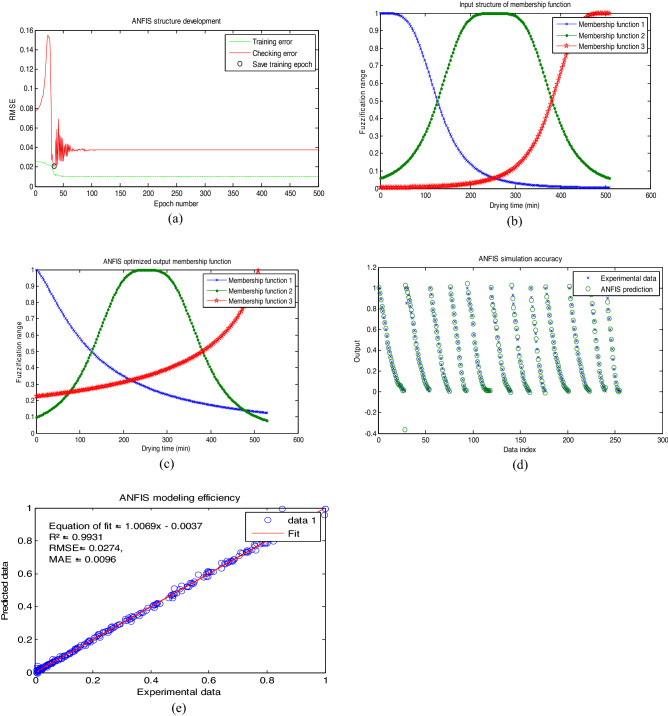
ANFIS performance plot.

In accordance, Fig. 6a–e represents the content of the most efficient ANFIS structure (three (3) numbers of gbellmf) developed and used for predictive modeling of the drying data in this study. Figure 6a–c showed the training part of the ANFIS structure while Fig. 6d,e showed the prediction part of the ANFIS structure. Figure 6a showed the training and checking error profile during the model development and informed that the training error decreased progressively throughout the training process. However, the checking error initial increased from zero epoch until 20 epochs before it decreased until 38 epochs as demarcated with the small circle after which the checking error increased again. This observation means that the ideal training epoch for this structure is 38, above which there exist a serious data over-fitting^32^. Data over-fitting implies data memorization rather than the required data feature understanding^42^.

During the course of ANFIS network structure development (i.e. training), the original form of the membership function undergoes refinement to sharpen its data approximation capability. The original form of the gbellmf used for predictive modeling in this study is represented in Fig. 6b while the refined form of the same gbellmf is represented in Fig. 6c. Comparing Fig. 6b,c, it is observed that the first and the third membership functions undergone significant refinement evident by change of shape while only the second membership function did not undergo refinement significant.

The simulation accuracy and modeling efficiency (parity plot) of the refined ANFIS structure that was used for the drying data approximation are shown in Fig. 6d,e. Figure 6d showed the one to one mapping of the ANFIS prediction of the drying data, and showed that significant portion of the experimental data were accurately mapped by the refined ANFIS structure. The parity plot in Fig. 6e showed that refined ANFIS structure has a high coefficient of determination (R^2^) that is close to unity while it has a root mean square error (RMSE) value of 0.0274 that is close to zero. Generally, a model having R^2^ value that is close to unity and RMSE value that is also close to zero is considered accurate^43^. Bousselma et al.^26^ derived R^2^ of 0.9995 in modeling the drying kinetics of pre-treated apricot with ANFIS. This result is important for process control during production.

### Sensitivity analysis

**Figure 7:**
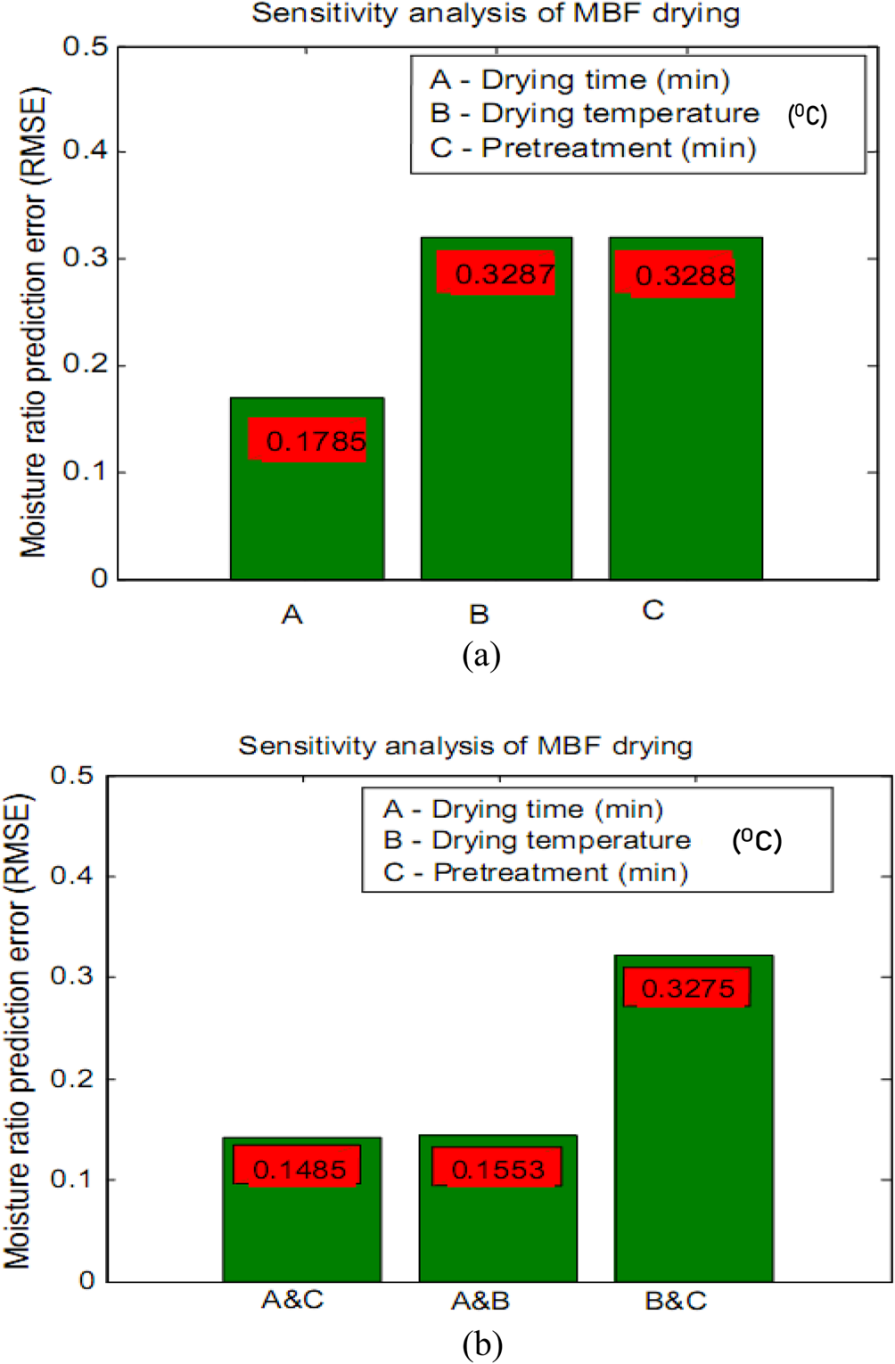
Sensitivity analysis of the drying factors at (**a**) individual contribution and (**b**) interactive contribution.

The individual and interactive contribution (as measured by prediction error) of drying process factors (drying time, drying temperature and pretreatment factor) to moisture ratio of MBF are represented in Fig. 7a,b.

The Fig. 7a showed that drying time contributed the least prediction error (0.1785) to the moisture ratio of MBF during drying. This was followed by drying temperature (0.3287) and pretreatment (0.3288). This means that moisture ratio of MBF during drying is most sensitive to drying time followed by drying temperature and pretreatment factor. This observation is logical because if given a sufficient drying time, a product undergoing moisture removal at any given drying temperature with or without pretreatment will get to its equilibrium moisture content. The idea of varied drying temperature and pretreatment type is to shorten the drying time. Figure 7a showed that the difference in the sensitivity of moisture ratio to drying temperature factor compared to pretreatment factor is however marginal.

Considering the sensitivity of moisture ratio of MBF to the interactive or combined drying factors, Fig. 7b showed that the drying time cum pretreatment factor interaction contributed prediction error of 0.1485 while drying time cum drying temperature factor interaction contributed 0.1553. In addition, drying temperature cum pretreatment interactively contributed 0.3275. This means that on factor interaction basis, moisture ratio of MBF is most sensitive to drying time cum pretreatment factor, followed by drying time cum drying temperature factor and lastly followed by drying temperature cum pretreatment factor. This result is important for decision-making and operational control during production.

## Conclusion

The effect of thermal pretreatment and drying temperature on the drying characteristics of MBF were investigated and modeled with ANFIS. The sensitivity of moisture ratio during drying to individual and interactive drying process factors were also investigated using ANFIS. These were aimed for drying equipment design and commercialization of MBF dried products. It was concluded from the results that MBF drying occurred in falling rate period only. The thermal pretreatment and drying temperature assisted in reducing the drying time significantly. The range of effective moisture diffusivities fall within the range reported in the literature and the best effective moisture diffusivity was observed in MBF sample that was blanched for 3 min. The activation energies also fall within the ones reported for agro-product in the literature. The unblanched MBF sample had the least activation energy and 5 min hot water blanched sample had the highest activation energy. Thermal pretreatments significantly reduced the total and specific energy consumption. ANFIS structure of 3—gbell membership function—38 epochs showed efficiency in modeling the multidimensional MBF drying data with high coefficient of determination value and can be useful in process controller design. Sensitivity analysis showed that moisture ratio was most sensitive to drying time on individual factor contribution level, and to drying time cum pretreatment factors on interactive factors contribution level. Therefore, drying time should be given the highest consideration when deciding on the moisture ratio of dried MBF product.

## Data Availability

All data used in this study are included in the report.
